# Supplementary material for: Understanding the underlying drivers of obesity in Africa: a scoping review protocol
Source: BMJ Open. 2020 Nov 11;10(11):e040940. doi: 10.1136/bmjopen-2020-040940 (PMC7661354; doi:10.1136/bmjopen-2020-040940)
Supplement: Author's manuscript [file bmjopen-2020-040940.draft_revisions.pdf]

BMJ Open is committed to open peer review. As part of this commitment we make the peer review history of every article we publish publicly available.

When an article is published we post the peer reviewers' comments and the authors' responses online. We also post the versions of the paper that were used during peer review. These are the versions that the peer review comments apply to.

The versions of the paper that follow are the versions that were submitted during the peer review process. They are not the versions of record or the final published versions. They should not be cited or distributed as the published version of this manuscript.

BMJ Open is an open access journal and the full, final, typeset and author-corrected version of record of the manuscript is available on our site with no access controls, subscription charges or pay-per-view fees (http://bmjopen.bmj.com).

If you have any questions on BMJ Open's open peer review process please email
info.bmjopen@bmj.com

BMJ Open

Understanding the underlying drivers of obesity in Africa: A scoping review protocol.

Journal:	*BMJ Open*
Manuscript ID	bmjopen-2020-040940
Article Type:	Protocol
Date Submitted by the Author:	27-May-2020
Complete List of Authors:	Jaca, Anelisa; South African Medical Research Council, Cochrane South Africa<br>Iwu, Chinwe; South African Medical Research Council, Cochrane South Africa<br>Durão, Solange; South African Medical Research Council, Cochrane South Africa<br>Onyango, Adelheid W.; World Health Organization, Department of Nutrition for Health and Development<br>Wiysonge, Charles; South African Medical Research Council, Cochrane South Africa
Keywords:	Health policy < HEALTH SERVICES ADMINISTRATION & MANAGEMENT, NUTRITION & DIETETICS, PUBLIC HEALTH

SCHOLARONE™
Manuscripts

I, the Submitting Author has the right to grant and does grant on behalf of all authors of the Work (as defined in the below author licence), an exclusive licence and/or a non-exclusive licence for contributions from authors who are: i) UK Crown employees; ii) where BMJ has agreed a CC-BY licence shall apply, and/or iii) in accordance with the terms applicable for US Federal Government officers or employees acting as part of their official duties; on a worldwide, perpetual, irrevocable, royalty-free basis to BMJ Publishing Group Ltd ("BMJ") its licensees and where the relevant Journal is co-owned by BMJ to the co-owners of the Journal, to publish the Work in this journal and any other BMJ products and to exploit all rights, as set out in our licence.

The Submitting Author accepts and understands that any supply made under these terms is made by BMJ to the Submitting Author unless you are acting as an employee on behalf of your employer or a postgraduate student of an affiliated institution which is paying any applicable article publishing charge ("APC") for Open Access articles. Where the Submitting Author wishes to make the Work available on an Open Access basis (and intends to pay the relevant APC), the terms of reuse of such Open Access shall be governed by a Creative Commons licence – details of these licences and which Creative Commons licence will apply to this Work are set out in our licence referred to above.

Other than as permitted in any relevant BMJ Author's Self Archiving Policies, I confirm this Work has not been accepted for publication elsewhere, is not being considered for publication elsewhere and does not duplicate material already published. I confirm all authors consent to publication of this Work and authorise the granting of this licence.

For peer review only

Understanding the underlying drivers of obesity in Africa: A scoping review protocol.

Anelisa Jaca[1], Chinwe J Iwu[2], Solange Durao[1], Adelheid W Onyango[3], Charles S Wiysonge[1,4,5]

[1] Cochrane South Africa, South African Medical Research Council, Cape Town, South Africa

[2] Department of Nursing, Faculty of Medicine and Health Sciences, Stellenbosch University, Cape Town, South Africa

[3] World Health Organization Regional Office for Africa, Brazzaville, Congo

[4] School of Public Health and Family Medicine, University of Cape Town, Cape Town, South Africa

[5] Department of Global Health, Stellenbosch University, Cape Town, South Africa

*Corresponding author: Dr Anelisa Jaca, Email: anelisaj@gmail.com

For peer review only

Abstract

Introduction: The worldwide prevalence of obesity and overweight has doubled since 1980, such that approximately a third of the world's population is reported as obese or overweight. Obesity rates have increased in all ages and both sexes irrespective of geographical area, ethnicity or socioeconomic status. Due to the high prevalence, related health consequences and costs of childhood and adult obesity, there is a need to comprehensively identify and assess the major underlying drivers of obesity and overweight in the African context.

Methods and Analysis: This scoping review will be carried out as per the methodological outline by Arksey and O'Malley. The search strategy will be developed and search performed in the Scopus and PubMed electronic databases. In the first search, we will identify concepts that are used as an equivalent to obesity and overweight. Subsequently, we will search for studies comprising of search terms on the underlying factors that drive the development of obesity and overweight. Lastly, we will check reference lists for additional publications. Abstracts and full-text studies will independently be screened by two authors.

Ethics and dissemination: The proposed study will generate evidence from published data and hence does not require ethics approval. Evidence generated from this review will be disseminated through journal publications and conference presentations.

Strengths and limitations of this study

- This scoping review will synthesize evidence on all factors related to the drivers of obesity in Africa.
- This study will employ a rigorous methodology which involves an established research framework, a search strategy and a selection process.
- Our review will potentially not include all research papers related to this theme area as some may not be accessible or published.

For peer review only

Introduction

Obesity is an intricate, multifactorial and preventable health issue currently affecting, together with overweight, over a third of the world's population.[1] The prevalence of obesity is seen across all ages, populations, ethnic groups and socioeconomic status.[2] Obesity was once considered a high-income country health problem but it is now also commonly seen in low- and middle-income countries.[3 4] In low-income countries, this condition is more predominant among middle-aged adults, especially women of childbearing between the ages of 15 and 49 years, while it affects individuals of all ages and genders in high-income countries.[5] In 2016, over 1.9 billion adults were overweight and of those individuals, more than 650 million were obese, worldwide.[6] Approximately 40 million children under the age of 5 years and 340 million adolescents were reported to be obese between the years 2016 and 2018, globally.[7] The number of children under the age of 5 years, suffering from obesity or overweight in Africa, was estimated at 9.5 million in the year 2018.[8] It is estimated that 20% of the world's adult population will be obese and 38% overweight by 2030 if this health issue is not dealt with.[1]

The World Health Organization (WHO) defines obesity as surplus of fat that can affect one's health. The commonly used measure of obesity is the body mass index (BMI), calculated by dividing the body weight in kilograms by the square of height in meters.[9] The WHO defines a normal BMI range as 18.5 to 24.9 suggesting that individuals with BMI greater than 25 kg/m2 and 30 kg/m2 are considered overweight and obese, respectively.[10] Obesity affects nearly all physiological functions of the body and hence increases the risk of developing conditions such as cardiovascular (e.g., heart failure) and metabolic (type 2 diabetes mellitus, hypertension and stroke) diseases.[10 11 12]

Obesity is predominantly associated with the overconsumption of foods with high sugar, calories and fats through the over consumption of processed, inexpensive and poor quality foods that are not nutrient dense.[13 15] The excess food that is consumed is changed into triglycerides which are stored in adipose tissues, hence increasing body fat and ultimately resulting in weight gain.[14].[15]

For peer review only

Additionally, the nutrition transition, which encompasses changes from predominantly plant-based diets to those predominantly meat-based, is highly associated with weight gain and illnesses related to it.[16 17] Therefore, healthy eating behaviors can play a major role in reducing the prevalence of obesity and its related diseases.[18]

Alongside the rise in consumption of processed foods there has also been an increase in sedentary behaviors in Africa.[19 20] This may have contributed to increased rates of obesity as weight gain is associated with a positive energy balance, i.e. calorie intake is greater than expenditure.[21] Literature has shown that obesity rates in adults are generally significantly higher in sedentary compared to active individuals.[22] A decrease in daily energy expenditure is associated with weight gain while high levels of physical activity significantly mitigate weight gain.[23 24] In addition to dietary and sedentary patterns, other contributing factors to the high prevalence of obesity include commonly used medications, namely, psychotropic medications, diabetic treatments, antihypertensives, steroid hormones and contraceptives.[25]

Study Rationale

There is growing acknowledgement that it will be challenging to practice healthy lifestyles until we understand the factors that drive and contribute to unhealthy behaviors. Factors including, diet, level of physical activity and age have been widely reported to influence obesity. Obesity increases with age which may be explained by decreases in physical activity and metabolic activity in older adults. The more we understand how these, or other factors drive obesity in Africa, the better we will be able to address it. Without proper understanding of the drivers of obesity in this setting, many individuals will not only suffer from obesity-related conditions but from costs sustained during treatment. Since obesity not only contributes to poor health but also affects people's psychological and social well-being, addressing it requires understanding and appreciating our biology, behavior, environment and culture. There is a need to better understand the various factors that motivate our diet and sedentary patterns, driving obesity. To date, no synthesized evidence exists that investigated the underlying drivers of obesity in Africa.

For peer review only

Study Objectives

The objective of this scoping review is to chart the existing evidence on the underlying drivers of obesity and overweight in the African context.

Methodology

A scoping review is used to gather emerging evidence in various fields with relevance to time, population (e.g., country or context), source (e.g., peer-reviewed or grey literature) and origin (e.g., healthcare discipline or academic field). This study design is suitable for addressing broad concepts, questions that seek to assess the effects of interventions to inform practice, policy and research. We will thus conduct a scoping review in order to map the crucial concepts and bring together the existing evidence on the underlying drivers of obesity in the African context. We will carry out this scoping review employing a predefined protocol as per the Arksey and O'Malley methodological outline.[26] This outline includes identifying the research question, searching for relevant studies, selecting studies, charting and collating data, summarizing and reporting results. This scoping review will be reported as per the Preferred Reporting Items for Systematic reviews and Meta-Analyses extension for Scoping Reviews (PRISMA-ScR) Checklist.[27]

Defining the research question

This scoping review will identify the drivers of obesity in the African setting. The research question: "What are the underlying drivers of obesity among children and adults in Africa" was framed using the Population, Exposure, Concept and Context (PECC) element (Table 1).

For peer review only

Table 1: PECC element for defining the eligibility criteria of the studies for the research question.

Population	Exposure	Concept	Context
Children (aged from 2 to 18 years old)<br>Adults (18 years and older)	Risk factors or drivers of obesity and overweight	Obesity (BMI greater than or equal 30 kg/m2)<br>Overweight (BMI greater than or equal to 25 kg/m2)	Africa

For peer review only

Inclusion criteria

We will include studies of any design that focus on drivers or risk factors of obesity. These drivers may include dietary factors (e.g., overconsumption of high-energy/low nutrient, processed and obesogenic foods), sedentary behaviors (e.g., physical inactivity or increased sedentary behaviors) and commonly used medications (e.g., psychotropic medications, diabetic treatments, antihypertensives, steroid hormones and contraceptives). Furthermore, the studies must have assessed and reported outcomes such as the type of food consumed, type of medication taken, sedentary behavioral patterns and prevalence of obesity and overweight. This review will include obese and overweight individuals of all ages, i.e., children (between ages 2 to 12 years), adolescents (13 to 17 years) and adults (18 years and older) and both genders (male and female). We will define overweight adults as those with a BMI greater than or equal to 25 kg/m^2 while those with a BMI greater than or equal to 30 kg/m^2 will be considered obese. In children between the ages of 5 and 19 years, overweight will be defined as a BMI >+1SD equivalent to 25 kg/m^2 while those with a BMI >+2SD equivalent to 30kg/m^2 will be deemed obese.[28]

Selection of studies relevant to the research question

Search strategy

We will conduct a comprehensive literature search in Scopus and PubMed databases. No language or date restrictions will be used. In our search, we will combine the key terms such as

“obesity OR obese OR overweightness OR overweight OR weight gain” (Table 2). The first 100 search results from each database will be reviewed by the researchers to assess validity of the search strategy. When agreement is reached about the initial search strategy, the first 200 abstracts will be screened by two authors (AJ and CJI) on concepts potentially eligible for inclusion in the second search step. In the event of full agreement between the two authors on potentially eligible concepts, AJ will screen the rest of the abstracts. In the event of disagreement, CJI will screen the abstracts until full agreement is reached. After all abstracts have been screened, AJ and CJI will discuss all potentially eligible search terms and select words to be included in the subsequent search step. After having selected the different search terms, AJ and CJI will develop a search query in PubMed and then apply it to other databases. Following the searches, the two authors will also search reference lists of relevant studies for other eligible articles.

Table 2: Initial search strategy developed for PubMed for the scoping review on the underlying drivers of obesity and overweight among children and adults in Africa.

Search #	Search Texts and Syntaxes
#1	obesity OR obese OR overweight OR body mass index OR fat OR fatness
#2	(((ALGERIA) OR (ANGOLA) OR (BENIN) OR (BOTSWANA) OR (BURKINA FASO) OR (BURUNDI) OR (CAMEROON) OR (CENTRAL AFRICAN REPUBLIC) OR (CHAD) OR (COMOROS) OR (CONGO) OR (DEMOCRATIC REPUBLIC CONGO) OR (DJIBOUTI) OR (EGYPT) OR ((EQUATORIAL GUINEA) OR "EQUATORIAL GUINEA") OR (ERITREA) OR (ETHIOPIA) OR (GABON)) OR ((GAMBIA) OR (GHANA) OR (GUINEA) OR ((GUINEA BISSAU) OR "GUINEA BISSAU") OR (IVORY COAST) OR ((COTE D'IVOIRE) OR "COTE D'IVOIRE") OR ((COTE IVOIRE) OR "COTE IVOIRE") OR (KENYA) OR (LESOTHO) OR (LIBERIA) OR (LIBYA) OR (LIBIA) OR (JAMAHIRIYA) OR (JAMAHIRYIA) OR (MADAGASCAR) OR (MALAWI) OR (MALI) OR (MAURITANIA) OR (MAURITIUS) OR (MOROCCO)) OR ((MOZAMBIQUE) OR (MOCAMBIQUE) OR (NAMIBIA) OR (NIGER) OR (NIGERIA) OR (REUNION) OR (RWANDA) OR ((SAO TOME) OR "SAO TOME") OR (SENEGAL) OR (SEYCHELLES) OR ((SIERRA LEONE) OR "SIERRA LEONE") OR (SOMALIA) OR ((SOUTH AFRICA) OR "SOUTH AFRICA") OR ((ST HELENA) OR "ST HELENA") OR (SUDAN) OR (SWAZILAND) OR ESWATINI OR (TANZANIA) OR (TANGANYIKA) OR (TOGO) OR (TUNISIA)) OR ((UGANDA) OR ((WESTERN SAHARA) OR "WESTERN SAHARA") OR (ZAIRE) OR (ZAMBIA) OR (ZIMBABWE) OR (AFRICA[MH]) OR (SOUTH* AND AFRICA*) OR (WEST* AND AFRICA*) OR (EAST* AND AFRICA*) OR (NORTH* AND AFRICA*) OR (CENTRAL* AND AFRICA*) OR (SUB SAHARAN AFRICA*) OR (SUBSAHARAN AFRICA*) OR (AFRICA*))) NOT (((GUINEA PIG*) OR "GUINEA PIG*") OR ((ASPERGILLUS NIGER) OR "ASPERGILLUS NIGER"))

For peer review only

#3	#1 AND #2

Screening and selection

Two review authors (AJ and CJI) will independently screen the titles and abstracts of articles identified in the search output for potentially eligible studies. The authors will obtain the full text articles for any study that is considered potentially eligible and then independently appraise the full text of each potentially eligible study and classify it as either included or excluded. We will provide reasons for excluding potentially eligible studies from the review. Disagreements among the two authors during the screening and study selection process will be resolved through discussion and arbitration by a third author.

Data charting and extraction

Data will be extracted independently by two authors (AJ and CJI) from the eligible studies and differences resolved by a third authors (CSW). Data extracted will comprise study characteristics (year the study was conducted/published, country of origin, study design, number of study arms and comparators); participants (children and adults); setting [African countries) and drivers or risk factors of obesity. These will include socio-economic (e.g., age, education level, employment, gender and income level), behavioural (e.g., physical inactivity, diet and alcohol consumption) and medication related drivers.

Patient and public involvement

This study will employ publicly available data and hence will not involve any patients and the public.

Collating, summarizing and reporting results of the review

A PRISMA flow diagram will be used to report the final number of included studies in the review. We will synthesize study findings using narrative descriptions based on subjects that emerge

For peer review only

from the extracted data. The results will be compared and consolidated through consensus between two authors (AJ and CJI) where both quantitative and qualitative aspects of research evidence will be addressed. We will discuss and present data obtained from obesity related outcomes reported in each study.

Expert consultation

In order to confirm our findings and interpretations, an expert in the field of nutrition and obesity will be approached for consultation.

Ethics and dissemination

The methodology applied to carry out this review will comprise reviewing and collecting evidence from openly available data; therefore, this study does not require ethical approval. This scoping review will be the first to collect evidence that has investigated the underlying drivers of obesity in Africa. By identifying gaps in the current body of literature, this study can influence forthcoming research on obesity in Africa. The results that will be generated from this review will be shared with the public through peer-reviewed publications and national and international conferences.

Contributorship statement

AJ wrote the first draft of the protocol. CJI, SD, AWO and CSW critically revised the intellectual content of the manuscript.

Competing interests

None declared

Funding

World Health Organization Region for Africa

Data sharing statement

The data that will be generated from this paper will be made publicly available.

For peer review only

References

1. Adela Hruby, PhD M, Frank B. Hu, MD, PhD M. The Epidemiology of Obesity: A Big Picture. Pharmacoeconomics. 2015;33(7):673–89.
2. Ells LJ, Rees K, Brown T, Mead E, Azevedo LAL, Mcgeechan GJ, et al. Interventions for treating children and adolescents with overweight and obesity : an overview of Cochrane reviews. Int J Obes. 2018;1823–33.
3. Bleich SN, Vercammen KA, Zatz LY, Frelier JM, Ebbeling CB, Peeters A. Review Interventions to prevent global childhood overweight and obesity : a systematic review. LANCET Diabetes Endocrinol. 2017;8587(4):1–15.
4. Laws R, Campbell KJ, Van Der Pligt P, Russell G, Ball K, Lynch J, et al. The impact of interventions to prevent obesity or improve obesity related behaviours in children (0-5 years) from socioeconomically disadvantaged and/or indigenous families: A systematic review. Vol. 14, BMC Public Health. 2014.
5. Hutchesson MJ, De Jonge Mulock Houwer M, Brown HM, Lim S, Moran LJ, Vincze L, et al. Supporting women of childbearing age in the prevention and treatment of overweight and obesity: A scoping review of randomized control trials of behavioral interventions. BMC Womens Health. 2020;20(1):1–15.
6. World Health Organization. Obesity and overweight [Internet]. Available from: https://www.who.int/news-room/fact-sheets/detail/obesity-and-overweight
7. World Health Organization. Obesity [Internet]. Available from: https://www.afro.who.int/health-topics/obesity
8. Das S, Hossain MZ, Nesa MK. Levels and trends in child malnutrition in Bangladesh. Asia-Pacific Popul J. 2009;24(2):51–78.
9. Chin S, Kahathuduwa CN, Binks M. Physical activity and obesity : what we know and what we need to know *. Obes Rev. 2016;17:1226–44.
10. Chooi YC, Ding C, Magkos F. The epidemiology of obesity. Metabolism. 2019;92:6–10.
11. Vecchié A, Dallegri F, Carbone F, Bonaventura A, Liberale L, Portincasa P, et al. European Journal of Internal Medicine Obesity phenotypes and their paradoxical association with cardiovascular diseases. Eur J Intern Med. 2018;48:6–17.
12. Seravalle G, Grassi G. Obesity and hypertension. Pharmacol Res. 2017;122:1–7.
13. Jacoby E, Grajeda R, Contreras A, Hospedales J. The epidemic of childhood obesity in the Americas must be stopped : Governmental and PAHO leadership are crucial. Int J Obes Suppl. 2013;3(S1):S15–7.
14. Mittendorfer B. Origins of metabolic complications in obesity: Adipose tissue and free fatty acid trafficking. Curr Opin Clin Nutr and Metab Care. 2011;14(6):535–41.
15. Harris J, Harris J. Nutrition transition in Zambia : Changing food supply , food prices , household consumption , diet and nutrition outcomes. Food Secur. 2019;371–87.
16. Fox A, Feng W, Asal V. What is driving global obesity trends ? Globalization or " modernization "? Glob Health. 2019;1–16.
17. Hills AP, Byrne NM, Lindstrom R, Hill JO. Small changes to diet and physical activity

For peer review only

behaviors for weight management. Obesity Facts. 2013;6:228–38.

18. Rush EC, Yan MR. Evolution not Revolution : Nutrition and Obesity. 2017;1–8.
19. Lien ASY, Tsai JL, Lee JT, Wu MY, Jiang Y Der, Yen HR. A Systematic Review and Meta-Analysis of the Effect of Lifestyle Modification on Metabolic Control in Overweight Children. Evidence-based Complement Altern Med. 2017;2017:1-12.
20. Scott A, Ejikeme CS, Clottey EN, Thomas JG. Obesity in sub-Saharan Africa: Development of an ecological theoretical framework. Health Promot Int. 2013;28(1):4–16.
21. Romieu I, Dossus L, Barquera S, Blottière HM, Franks PW, Gunter M, et al. Energy balance and obesity: what are the main drivers? Cancer Causes Control. 2017;28(3):247–58.
22. Nantel J, Mathieu M. Physical Activity and Obesity: Biomechanical and Physiological Key Concepts. J. Obes. 2015;2011:1-10.
23. Liu Z, Xu H, Wen L, Peng Y, Lin L, Zhou S, et al. A systematic review and meta-analysis of the overall effects of school-based obesity prevention interventions and effect differences by intervention components. Int J Behav Nutr Phy. 2019;1–12.
24. Wiklund P. The role of physical activity and exercise in obesity and weight management : Time for critical appraisal. J Sport Heal Sci. 2016;5(2):151–4.
25. Wright SM, Aronne LJ. Causes of obesity. Abdom Imaging. 2012;37:730–2.
26. Arksey H, O'Malley L. Scoping studies: towards a methodological framework. Int J Soc. 2005;8(1):19-32.
27. Tricco AC, Lillie E, Zarin W, Brien KKO, Colquhoun H, Levac D, et al. R ESEARCH AND R EPORTING M ETHODS PRISMA Extension for Scoping Reviews (PRISMA-ScR): Checklist and Explanation. Ann Intern Med. 2018;169(7):467–73.
28. World Health Organization. Growth reference [Internet]. Available from https://www.who.int/growthref/en/

For peer review only

BMJ Open

Understanding the underlying drivers of obesity in Africa: A scoping review protocol.

Journal:	*BMJ Open*
Manuscript ID	bmjopen-2020-040940.R1
Article Type:	Protocol
Date Submitted by the Author:	11-Aug-2020
Complete List of Authors:	Jaca, Anelisa; South African Medical Research Council, Cochrane South Africa<br>Iwu, Chinwe; South African Medical Research Council, Cochrane South Africa<br>Durão, Solange; South African Medical Research Council, Cochrane South Africa<br>Onyango, Adelheid W.; World Health Organization, Department of Nutrition for Health and Development<br>Wiysonge, Charles; South African Medical Research Council, Cochrane South Africa
**Primary Subject Heading**:	Nutrition and metabolism
Secondary Subject Heading:	Nutrition and metabolism, Evidence based practice, Global health, Health policy
Keywords:	Health policy < HEALTH SERVICES ADMINISTRATION & MANAGEMENT, NUTRITION & DIETETICS, PUBLIC HEALTH, Nutritional support < GASTROENTEROLOGY

SCHOLARONE™
Manuscripts

I, the Submitting Author has the right to grant and does grant on behalf of all authors of the Work (as defined in the below author licence), an exclusive licence and/or a non-exclusive licence for contributions from authors who are: i) UK Crown employees; ii) where BMJ has agreed a CC-BY licence shall apply, and/or iii) in accordance with the terms applicable for US Federal Government officers or employees acting as part of their official duties; on a worldwide, perpetual, irrevocable, royalty-free basis to BMJ Publishing Group Ltd ("BMJ") its licensees and where the relevant Journal is co-owned by BMJ to the co-owners of the Journal, to publish the Work in this journal and any other BMJ products and to exploit all rights, as set out in our licence.

The Submitting Author accepts and understands that any supply made under these terms is made by BMJ to the Submitting Author unless you are acting as an employee on behalf of your employer or a postgraduate student of an affiliated institution which is paying any applicable article publishing charge ("APC") for Open Access articles. Where the Submitting Author wishes to make the Work available on an Open Access basis (and intends to pay the relevant APC), the terms of reuse of such Open Access shall be governed by a Creative Commons licence – details of these licences and which Creative Commons licence will apply to this Work are set out in our licence referred to above.

Other than as permitted in any relevant BMJ Author's Self Archiving Policies, I confirm this Work has not been accepted for publication elsewhere, is not being considered for publication elsewhere and does not duplicate material already published. I confirm all authors consent to publication of this Work and authorise the granting of this licence.

For peer review only

Understanding the underlying drivers of obesity in Africa: A scoping review protocol.

Anelisa Jaca[1], Chinwe J Iwu[2], Solange Durao[1], Adelheid W Onyango[3], Charles S Wiysonge[1,4,5]

[1] Cochrane South Africa, South African Medical Research Council, Cape Town, South Africa

[2] Department of Nursing, Faculty of Medicine and Health Sciences, Stellenbosch University, Cape Town, South Africa

[3] World Health Organization Regional Office for Africa, Brazzaville, Congo

[4] School of Public Health and Family Medicine, University of Cape Town, Cape Town, South Africa

[5] Department of Global Health, Stellenbosch University, Cape Town, South Africa

*Corresponding author: Dr Anelisa Jaca, Email: anelisaj@gmail.com

For peer review only

For peer review only - http://bmjopen.bmj.com/site/about/guidelines.xhtml

Abstract

Introduction: The worldwide prevalence of obesity and overweight has doubled since 1980, such that approximately a third of the world's population is reported as obese or overweight. Obesity rates have increased in all ages and both sexes irrespective of geographical area, ethnicity or socioeconomic status. Due to the high prevalence, related health consequences and costs of childhood and adult obesity, there is a need to comprehensively identify and assess the major underlying drivers of obesity and overweight in the African context.

Methods and Analysis: This scoping review will be carried out as per the methodological outline by Arksey and O'Malley. The search strategy will be developed and search performed in the Scopus and PubMed electronic databases. In the first search, we will identify concepts that are used as an equivalent to obesity and overweight. Subsequently, we will search for studies comprising of search terms on the underlying factors that drive the development of obesity and overweight. Lastly, we will check reference lists for additional publications. Abstracts and full-text studies will independently be screened by two authors.

Ethics and dissemination: The proposed study will generate evidence from published data and hence does not require ethics approval. Evidence generated from this review will be disseminated through journal publications and conference presentations.

Strengths and limitations of this study

- This scoping review will synthesize evidence on all factors related to the drivers of obesity in Africa.
- This study will employ a rigorous methodology which involves an established research framework, a search strategy and a selection process.
- Our review will potentially not include all research papers related to this theme area as some may not be accessible or published.

For peer review only - http://bmjopen.bmj.com/site/about/guidelines.xhtml

Introduction

Obesity is an intricate, multifactorial and preventable health issue currently affecting, together with overweight, over a third of the world's population.[1] The prevalence of obesity is seen across all ages, populations, ethnic groups and socioeconomic status.[2] Obesity was once considered a high-income country health problem but it is now also commonly seen in low- and middle-income countries.[3 4] In low-income countries, this condition is more predominant among middle-aged adults, especially women of childbearing between the ages of 15 and 49 years, while it affects individuals of all ages and genders in high-income countries.[5] In 2016, over 1.9 billion adults were overweight and of those individuals, more than 650 million were obese, worldwide.[6] Approximately 40 million children under the age of 5 years and 340 million adolescents were reported to be obese between the years 2016 and 2018, globally.[7] The number of children under the age of 5 years, suffering from obesity or overweight in Africa, was estimated at 9.5 million in the year 2018.[8] It is estimated that 20% of the world's adult population will be obese and 38% overweight by 2030 if this health issue is not dealt with.[1]

The World Health Organization (WHO) defines obesity as surplus of fat that can affect one's health. The commonly used measure of obesity is the body mass index (BMI), calculated by dividing the body weight in kilograms by the square of height in meters.[9] The WHO defines a normal BMI range as 18.5 to 24.9 suggesting that individuals with BMI greater than 25 kg/m2 and 30 kg/m2 are considered overweight and obese, respectively.[10] Obesity affects nearly all physiological functions of the body and hence increases the risk of developing conditions such as cardiovascular (e.g., heart failure) and metabolic (type 2 diabetes mellitus, hypertension and stroke) diseases.[10 11 12]

Obesity is predominantly associated with the overconsumption of foods with high sugar, calories and fats through the over consumption of processed, inexpensive and poor quality foods that are not nutrient dense.[13] The excess food that is consumed is changed into triglycerides which are stored in adipose tissues, hence increasing body fat and ultimately resulting in weight gain.[14].[15]

For peer review only - http://bmjopen.bmj.com/site/about/guidelines.xhtml

Additionally, the nutrition transition, which encompasses changes from predominantly plant-based diets to those predominantly meat-based, is highly associated with weight gain and illnesses related to it.[16 17] Therefore, healthy eating behaviors can play a major role in reducing the prevalence of obesity and its related diseases.[18]

Alongside the rise in consumption of processed foods there has also been an increase in sedentary behaviors in Africa.[19 20] This may have contributed to increased rates of obesity as weight gain is associated with a positive energy balance, i.e. calorie intake is greater than expenditure.[21] Literature has shown that obesity rates in adults are generally significantly higher in sedentary compared to active individuals.[22] A decrease in daily energy expenditure is associated with weight gain while high levels of physical activity significantly mitigate weight gain.[23 24] Physical activity is defined as any bodily movement that demands energy use, e.g., through walking, cycling, sport activities and doing house chores. On the other hand, physical inactivity or sedentary behavior is a lifestyle identified by an energy expenditure less than 1.5 metabolic equivalents (METs) while in a sitting, reclining or lying position [25]. In addition to dietary and sedentary patterns, other contributing factors to the high prevalence of obesity include smoking, alcohol consumption, urbanization and commonly used medications, namely, psychotropic medications, diabetic treatments, antihypertensives, steroid hormones and contraceptives.[26]

Study Rationale

There is growing acknowledgement that it will be challenging to practice healthy lifestyles until we understand the factors that drive and contribute to unhealthy behaviors. Factors including, diet, level of physical activity and age have been widely reported to influence obesity. Obesity increases with age which may be explained by decreases in physical activity and metabolic activity in older adults. The more we understand how these, or other factors drive obesity in Africa, the better we will be able to address it. Without proper understanding of the drivers of obesity in this setting, many individuals will not only suffer from obesity-related conditions but from costs sustained during treatment. Since obesity not only contributes to poor health but also affects people's psychological and social well-being, addressing it requires understanding and

For peer review only - http://bmjopen.bmj.com/site/about/guidelines.xhtml

appreciating our biology, behavior, environment and culture. There is a need to better understand the various factors that motivate our diet and sedentary patterns, driving obesity. To date, no synthesized evidence exists that investigated the underlying drivers of obesity in Africa. We will therefore conduct a narrative synthesis of the underlying drivers of obesity in the African setting.

Study Objectives

The objective of this scoping review is to chart the existing evidence on the underlying drivers of obesity and overweight in the African context.

Methodology

A scoping review is used to gather emerging evidence in various fields with relevance to time, population (e.g., country or context), source (e.g., peer-reviewed or grey literature) and origin (e.g., healthcare discipline or academic field). This study design is suitable for addressing broad concepts, questions that seek to assess the effects of interventions to inform practice, policy and research. We will thus conduct a scoping review, from the 1st of September 2020 to the 26th of February 2021, in order to map the crucial concepts and bring together the existing evidence on the underlying drivers of obesity in the African context. We will carry out this scoping review employing a predefined protocol as per the Arksey and O'Malley methodological outline.[27] This outline includes identifying the research question, searching for relevant studies, selecting studies, charting and collating data, summarizing and reporting results. This scoping review will be reported as per the Preferred Reporting Items for Systematic reviews and Meta-Analyses extension for Scoping Reviews (PRISMA-ScR) Checklist.[28]

Defining the research question

This scoping review will identify the drivers of obesity in the African setting. The research question: "What are the underlying drivers of obesity among children and adults in Africa" was framed using the Population, Exposure, Concept and Context (PECC) element (Table 1).

For peer review only - http://bmjopen.bmj.com/site/about/guidelines.xhtml

Table 1: PECC element for defining the eligibility criteria of the studies for the research question.

Population	Exposure	Concept	Context
Children (between ages 2 to 12 years), adolescents (13 to 17 years), young adults (18 years to 35 years), older adults (36 to 55 years) and the elderly (56 year and older).	Risk factors or drivers of obesity and overweight	Obesity (BMI greater than or equal 30 kg/m2)<br>Overweight (BMI greater than or equal to 25 kg/m2)	Africa

Inclusion criteria

We will include studies of any design that focus on drivers or risk factors of obesity. These drivers may include dietary factors (e.g., overconsumption of high-energy/low nutrient, processed and obesogenic foods), sedentary behaviors (e.g., physical inactivity or increased sedentary behaviors), smoking status, alcohol consumption, urbanization, and commonly used medications (e.g., psychotropic medications, diabetic treatments, antihypertensives, steroid hormones and contraceptives). Furthermore, the studies must have assessed and reported outcomes such as the type of food consumed, type of medication taken, sedentary behavioral patterns and prevalence of obesity and overweight. This review will include obese and overweight individuals of all ages, i.e., children (between ages 2 to 12 years), adolescents (13 to 17 years), young adults(18 to 35 years), older adults (36 to 55 years) and the elderly (56 years and older) and both genders (male and female). We will define overweight adults as those with a BMI greater than or equal to 25 kg/m^2 while those with a BMI greater than or equal to 30 kg/m^2 will be considered

For peer review only

For peer review only - http://bmjopen.bmj.com/site/about/guidelines.xhtml

obese. In children between the ages of 5 and 19 years, overweight will be defined as a BMI >+1SD equivalent to 25 kg/m ^{2} while those with a BMI >+2SD equivalent to 30kg/m^2 will be deemed obese.[29]

Search #	Search Texts and Syntaxes
#1	obesity OR obese OR overweight OR body mass index OR fat OR fatness
#2	physical inactivity OR physically inactive OR sedentar*

Selection of studies relevant to the research question

Search strategy

We will conduct a comprehensive literature search in Scopus and PubMed databases. No language or date restrictions will be used. In our search, we will combine the key terms such as "obesity OR obese OR overweightness OR overweight OR weight gain" AND physical inactivity OR physically inactive OR sedentar* (Table 2). The first 100 search results from each database will be reviewed by the researchers to assess validity of the search strategy. When agreement is reached about the initial search strategy, the first 200 abstracts will be screened by two authors (AJ and CJI) on concepts potentially eligible for inclusion in the second search step. In the event of full agreement between the two authors on potentially eligible concepts, AJ will screen the rest of the abstracts. In the event of disagreement, CJI will screen the abstracts until full agreement is reached. After all abstracts have been screened, AJ and CJI will discuss all potentially eligible search terms and select words to be included in the subsequent search step. After having selected the different search terms, AJ and CJI will develop a search query in PubMed and then apply it to other databases. Following the searches, the two authors will also search reference lists of relevant studies for other eligible articles.

Table 2: Initial search strategy developed for PubMed for the scoping review on the underlying drivers of obesity and overweight among children and adults in Africa.

For peer review only - http://bmjopen.bmj.com/site/about/guidelines.xhtml

#3	(((ALGERIA) OR (ANGOLA) OR (BENIN) OR (BOTSWANA) OR (BURKINA FASO) OR (BURUNDI) OR (CAMEROON) OR (CENTRAL AFRICAN REPUBLIC) OR (CHAD) OR (COMOROS) OR (CONGO) OR (DEMOCRATIC REPUBLIC CONGO) OR (DJIBOUTI) OR (EGYPT) OR ((EQUATORIAL GUINEA) OR "EQUATORIAL GUINEA") OR (ERITREA) OR (ETHIOPIA) OR (GABON)) OR ((GAMBIA) OR (GHANA) OR (GUINEA) OR ((GUINEA BISSAU) OR "GUINEA BISSAU") OR (IVORY COAST) OR ((COTE D'IVOIRE) OR "COTE D'IVOIRE") OR ((COTE IVOIRE) OR "COTE IVOIRE") OR (KENYA) OR (LESOTHO) OR (LIBERIA) OR (LIBYA) OR (LIBIA) OR (JAMAHIRIYA) OR (JAMAHIRYIA) OR (MADAGASCAR) OR (MALAWI) OR (MALI) OR (MAURITANIA) OR (MAURITIUS) OR (MOROCCO)) OR ((MOZAMBIQUE) OR (MOCAMBIQUE) OR (NAMIBIA) OR (NIGER) OR (NIGERIA) OR (REUNION) OR (RWANDA) OR ((SAO TOME) OR "SAO TOME") OR (SENEGAL) OR (SEYCHELLES) OR ((SIERRA LEONE) OR "SIERRA LEONE") OR (SOMALIA) OR ((SOUTH AFRICA) OR "SOUTH AFRICA") OR ((ST HELENA) OR "ST HELENA") OR (SUDAN) OR (SWAZILAND) OR ESWATINI OR (TANZANIA) OR (TANGANYIKA) OR (TOGO) OR (TUNISIA)) OR ((UGANDA) OR ((WESTERN SAHARA) OR "WESTERN SAHARA") OR (ZAIRE) OR (ZAMBIA) OR (ZIMBABWE) OR (AFRICA[MH]) OR (SOUTH* AND AFRICA*) OR (WEST* AND AFRICA*) OR (EAST* AND AFRICA*) OR (NORTH* AND AFRICA*) OR (CENTRAL* AND AFRICA*) OR (SUB SAHARAN AFRICA*) OR (SUBSAHARAN AFRICA*) OR (AFRICA*))) NOT (((GUINEA PIG*) OR "GUINEA PIG*") OR ((ASPERGILLUS NIGER) OR "ASPERGILLUS NIGER"))
#4	#1 AND #2 AND #3

Screening and selection

Two review authors (AJ and CJI) will independently screen the titles and abstracts of articles identified in the search output for potentially eligible studies. The authors will obtain the full text articles for any study that is considered potentially eligible and then independently appraise the full text of each potentially eligible study and classify it as either included or excluded. We will provide reasons for excluding potentially eligible studies from the review. Disagreements among

the two authors during the screening and study selection process will be resolved through discussion and arbitration by a third author.

Data charting and extraction

Data will be extracted independently by two authors (AJ and CJI) from the eligible studies and differences resolved by a third authors (CSW). Data extracted will comprise study characteristics (year the study was conducted/published, country of origin, study design, number of study arms and comparators); participants (children and adults); setting [African countries) and drivers or risk factors of obesity. These will include demographic (age and gender), environmental (suburb and rural areas), food availability (high calories and energy dense food), socio-economic (education level, employment status and income level), behavioural (e.g., physical inactivity, diet and alcohol consumption) and medication related drivers.

Patient and public involvement

This study will employ publicly available data and hence will not involve any patients and the public.

Collating, summarizing and reporting results of the review

A PRISMA flow diagram will be used to report the final number of included studies in the review. We will synthesize study findings using narrative descriptions based on subjects that emerge from the extracted data. The results will be compared and consolidated through consensus between two authors (AJ and CJI) where both quantitative and qualitative aspects of research evidence will be addressed. We will discuss and present data obtained from obesity related outcomes reported in each study.

Expert consultation

In order to confirm our findings and interpretations, an expert in the field of nutrition and obesity will be approached for consultation.

For peer review only - http://bmjopen.bmj.com/site/about/guidelines.xhtml

Ethics and dissemination

The methodology applied to carry out this review will comprise reviewing and collecting evidence from openly available data; therefore, this study does not require ethical approval. This scoping review will be the first to collect evidence that has investigated the underlying drivers of obesity in Africa. By identifying gaps in the current body of literature, this study can influence forthcoming research on obesity in Africa. The results that will be generated from this review will be shared with the public through peer-reviewed publications and national and international conferences.

Contributorship statement

AJ wrote the first draft of the protocol. CJI, SD, AWO and CSW critically revised the intellectual content of the manuscript.

Competing interests

None declared

Funding

World Health Organization Region for Africa (grant number: N/A).

Data sharing statement

The data that will be generated from this paper will be made publicly available.

References

1. Adela Hruby, PhD M, Frank B. Hu, MD, PhD M. The Epidemiology of Obesity: A Big Picture. Pharmacoeconomics. 2015;33(7):673–89.
2. Ells LJ, Rees K, Brown T, Mead E, Azevedo LAL, Mcgeechan GJ, et al. Interventions for treating children and adolescents with overweight and obesity : an overview of Cochrane reviews. Int J Obes. 2018;1823–33.
3. Bleich SN, Vercammen KA, Zatz LY, Frelier JM, Ebbeling CB, Peeters A. Review Interventions to prevent global childhood overweight and obesity : a systematic review. LANCET Diabetes Endocrinol. 2017;8587(4):1–15.
4. Laws R, Campbell KJ, Van Der Pligt P, Russell G, Ball K, Lynch J, et al. The impact of

For peer review only

For peer review only - http://bmjopen.bmj.com/site/about/guidelines.xhtml

interventions to prevent obesity or improve obesity related behaviours in children (0-5 years) from socioeconomically disadvantaged and/or indigenous families: A systematic review. Vol. 14, BMC Public Health. 2014.

5. Hutchesson MJ, De Jonge Mulock Houwer M, Brown HM, Lim S, Moran LJ, Vincze L, et al. Supporting women of childbearing age in the prevention and treatment of overweight and obesity: A scoping review of randomized control trials of behavioral interventions. BMC Womens Health. 2020;20(1):1–15.

6. World Health Organization. Obesity and overweight [Internet]. [cited 2020 Apr 3]. Available from: https://www.who.int/news-room/fact-sheets/detail/obesity-and-overweight

7. Wordld Health Organization. Obesity [Internet]. Available from: https://www.afro.who.int/health-topics/obesity

8. Das S, Hossain MZ, Nesa MK. Levels and trends in child malnutrition in Bangladesh. Asia-Pacific Popul J. 2009;24(2):51–78.

9. Chin S, Kahathuduwa CN, Binks M. Physical activity and obesity : what we know and what we need to know *. 2016;(December):1226–44.

10. Chooi YC, Ding C, Magkos F. The epidemiology of obesity. Metabolism. 2019;92:6–10.

11. Vecchié A, Dallegri F, Carbone F, Bonaventura A, Liberale L, Portincasa P, et al. European Journal of Internal Medicine Obesity phenotypes and their paradoxical association with cardiovascular diseases. Eur J Intern Med. 2018;48(October 2017):6–17.

12. Seravalle G, Grassi G. Obesity and hypertension. Pharmacol Res. 2017;122:1–7.

13. Jacoby E, Grajeda R, Contreras A, Hospedales J. The epidemic of childhood obesity in the Americas must be stopped : Governmental and PAHO leadership are crucial. Int J Obes Suppl. 2013;3(S1):S15–7.

14. Mittendorfer B. Origins of metabolic complications in obesity: Adipose tissue and free fatty acid trafficking. Vol. 14, Current Opinion in Clinical Nutrition and Metabolic Care. 2011. p. 535–41.

15. Harris J, Harris J. Nutrition transition in Zambia : Changing food supply , food prices , household consumption , diet and nutrition outcomes. 2019;371–87.

16. Fox A, Feng W, Asal V. What is driving global obesity trends ? Globalization or “ modernization ” ? 2019;1–16.

17. Hills AP, Byrne NM, Lindstrom R, Hill JO. Small changes to diet and physical activity behaviors for weight management. Vol. 6, Obesity Facts. 2013. p. 228–38.

18. Rush EC, Yan MR. Evolution not Revolution : Nutrition and Obesity. 2017;1–8.

19. Lien ASY, Tsai JL, Lee JT, Wu MY, Jiang Y Der, Yen HR. A Systematic Review and Meta-Analysis of the Effect of Lifestyle Modification on Metabolic Control in Overweight Children. Evidence-based Complement Altern Med. 2017;2017.

20. Scott A, Ejikeme CS, Clottey EN, Thomas JG. Obesity in sub-Saharan Africa: Development of an ecological theoretical framework. Health Promot Int. 2013;28(1):4–16.

21. Romieu I, Dossus L, Barquera S, Blottière HM, Franks PW, Gunter M, et al. Energy balance and obesity: what are the main drivers? Cancer Causes Control. 2017;28(3):247–58.

22. Nantel J, Mathieu M. Physical Activity and Obesity : Biomechanical and Physiological Key Concepts. 2015;2011.

23. Liu Z, Xu H, Wen L, Peng Y, Lin L, Zhou S, et al. A systematic review and meta-analysis of

For peer review only - http://bmjopen.bmj.com/site/about/guidelines.xhtml

the overall effects of school-based obesity prevention interventions and effect differences by intervention components. 2019;1–12.
24. Wiklund P. The role of physical activity and exercise in obesity and weight management : Time for critical appraisal. J Sport Heal Sci. 2016;5(2):151–4.
25. van der Ploeg HP, Hillsdon M. Is sedentary behaviour just physical inactivity by another name? Int J Behav Nutr Phys Act. 2017;14(1):1–8.
26. Wright SM, Aronne LJ. Causes of obesity. 2012;(March):730–2.
27. Özsu U. De-territorializing and Re-territorializing Lotus: Sovereignty and Systematicity as Dialectical Nation-Building in Early Republican Turkey. Leiden J Int Law. 2009;22(1):29–49.
28. Tricco AC, Lillie E, Zarin W, Brien KKO, Colquhoun H, Levac D, et al. R ESEARCH AND R EPORTING M ETHODS PRISMA Extension for Scoping Reviews (PRISMA-ScR): Checklist and Explanation. 2018;
29. World Health Organization. Growth reference 5-19 years. 2020;19.

For peer review only

For peer review only - http://bmjopen.bmj.com/site/about/guidelines.xhtml
